# Irisin Attenuates Myocardial Ischemia/Reperfusion Injury and Improves Mitochondrial Function Through AMPK Pathway in Diabetic Mice

**DOI:** 10.3389/fphar.2020.565160

**Published:** 2020-09-11

**Authors:** Chao Xin, Zheng Zhang, Guojie Gao, Liping Ding, Chao Yang, Chengzhu Wang, Yanjun Liu, Yufei Guo, Xueqing Yang, Lijuan Zhang, Lina Zhang, Yi Liu, Zhitao Jin, Ling Tao

**Affiliations:** ^1^Department of Cardiology, PLA Rocket Force Characteristic Medical Center, Beijing, China; ^2^Department of Cardiology, Xijing Hospital, Air Force Medical University, Xi’an, China

**Keywords:** irisin, ischemia/reperfusion, mitochondria, AMPK, diabetes

## Abstract

**Aims:**

Several recent reports have shown irisin protects the heart against ischemia/reperfusion injury. However, the effect of irisin on I/R injury in diabetic mice has not been described. The present study was designed to investigate the role of irisin in myocardial ischemia-reperfusion (MI/R) injury in diabetic mice.

**Methods:**

A mouse model of diabetes was established by feeding wild type or gene-manipulated adult male mice with a high-fat diet. All the mice received intraperitoneal injection of irisin or PBS. Thirty minutes after injection, mice were subjected to 30 min of myocardial ischemia followed by 3h (for cell apoptosis and protein determination), 24 h (for infarct size and cardiac function).

**Results:**

Knock-out of gene FNDC5 augmented MI/R injury in diabetic mice, while irisin treatment attenuated MI/R injury, improved cardiac function, cellular ATP biogenetics, mitochondria potential, and impaired mitochondrion-related cell death. More severely impaired AMPK pathway was observed in diabetic FNDC5^-/-^ mice received MI/R. Knock-out of gene AMPK blocks the beneficial effects of irisin on MI/R injury, cardiac function, cellular ATP biogenetics, mitochondria potential, and mitochondrion-related cell death.

**Conclusions:**

Our present study demonstrated that irisin improves the mitochondria function and attenuates MI/R injury in diabetic mice through AMPK pathway.

## Introduction

Diabetes is one of the most frequent risk factors of cardiovascular diseases (Ye et al., 2011). It has been demonstrated that compared with normal animals, MI/R-induced injuries occur more often in diabetic animals than in normal controls (Yue et al., 2005). These data indicate that diabetes enhances susceptibility to MI/R injury. However, treatments for MI/R injury in diabetic patients nowadays are not satisfactory.

Irisin, regulated by PPARγ coactivatou 1 alpha (PGC1-α), is a recently identified myokine inducing the browning of white adipose tissue, and mediating the beneficial effects of exercise and predominantly controlling metabolism of energy, glucose and lipid (Bostrom et al., 2012). It has been demonstrated to reduce oxidative stresses and apoptosis in several models (Park et al., 2015; Zhu et al., 2015). Our previous research has indicated that irisin could improve glucose and fatty acid metabolism (Xin et al., 2016), which suggests irisin could be used as a therapeutic tool for metabolic disorders and cardiovascular diseases.

Irisin plays an important role in protecting the heart against I/R injury *in vitro* perfused heart by regulating mitochondrial apoptosis (Fan et al., 2020), while whether irisin protects the diabetic heart from MI/R injury and its mechanism remain unknown. It is essential to define whether irisin protects heart from MI/R injury in diabetes. Thus, irisin could be developed as a novel strategy in the treatment of cardiovascular disease in diabetes.

Mitochondrial homeostasis has been found to be associated with hyperglycemia stress. (Sharafati-Chaleshtori et al., 2017) Several studies show that mitochondrial dysfunction occurs in the hearts of humans with diabetes (Bugger and Abel, 2010; Watanabe et al., 2010). Besides, mitochondria play an important role in the occurrence of apoptosis. Apoptosis is now considered to be one of the main pathophysiological mechanisms of MI/R injury (Cai et al., 2002; Badalzadeh et al., 2012). I/R induces apoptosis by down-regulating pro-death parameters level, up-regulating pro-survival proteins level and activating caspases. Myocardial apoptosis in the diabetic condition may induce more severe cardiac dysfunction and dysrhythmia (Badalzadeh et al., 2015).

AMP-activated protein kinase (AMPK) is a serine-threonine kinase acting as an energy sensor and can be activated by hypoxia stress (Tian and Balschi, 2006). Activation of AMPK signals down-regulates ATP utilizing pathways and up-regulates ATP-generating pathways when cellular energy is depleted, and thus restores energy homeostasis (Dolinsky and Dyck, 2006; Young, 2008). It has been demonstrated that AMPK plays an essential role in irisin’s metabolic regulatory, vasculoprotective actions, and cardioprotective actions. The role of AMPK in irisin’s anti-ischemic and cardioprotective action in diabetes remains understood.

Therefore, the aims of the present study were to determine 1) whether exogenous irisin supplementation could attenuate MI/R injury in diabetic mice, 2) whether irisin improves mitochondria function and suppresses mitochondrial apoptosis under MI/R injury, 3) whether irisin exerts its effects in an AMPK-dependent fashion.

## Materials and Methods

### Animals and Experimental Protocols

All animal experiments were performed according to the National Institutes of Health Guidelines on the Use of Laboratory Animals, and were approved by the Fourth Military Medical University Committee on Animal Care. FNDC- knockout (FNDC5^-/-^) and Cardiac-specific AMPK knockout (AMPK^-/-^) mice of both sexes were generated and backcrossed as described previously. Age (8–10 weeks) and sexmatched C57BL6/J littermates [wild-type (WT) Experimental Animal Center, Fourth Military Medical University] were used as controls. All the mice maintained in a temperature-controlled barrier facility with free access to food and water and a 12-hour light/dark cycle. Type 2 diabetes model was established as described. All the diabetic mice (including diabetic FNDC5^-/-^, AMPK^-/-^ and WT mice) were randomized to receive sham-operation or myocardial I/R as described above. All surgical procedures were performed under anesthesia to avoid experimental stresses.

All the diabetic mice were administered with vehicle (PBS) or irisin (0.5 μg/g, irisin/body weight, Abnova, Taiwan, China)(a dose determined from previous report (Zhang et al., 2014) before receiving sham-operation or myocardial I/R. Thirty minutes after injection, mice were subjected to 30 min of myocardial ischemia followed by 3h (for cell apoptosis and protein tests), 24 h (for infarct size and cardiac function).

### Induction of Type 2 Diabetes

Type 2 diabetes model was established by feeding animals with high-fat diet (D12492, Research Diets, Inc; NJ; USA) containing (kcal) 20% protein, 20% carbohydrate, and 60% fat for 10 weeks. Control mice were fed with chow diet (D12450B, Research Diets, Inc; NJ; USA) containing (kcal) 20% protein, 70% carbohydrate, and 10% fat. Blood glucose and body weight were measured, and a diabetic condition was confirmed on the basis of a non-fasting blood glucose level of ≥200 mg/dl.

### Induction of MI/R

Mice were anesthetized with 2% isoflurane, and myocardial infarction (MI) was produced by temporarily exteriorizing the heart *via* a left thoracic incision and placing a 6-0 silk suture slipknot around the left anterior descending coronary artery. After 30 min of MI, the slipknot was released, and the myocardium was reperfused for 3 (for myocardial apoptosis evaluation) or 24 h (for cardiac function and infarct size determination). Sham-operated control mice (sham MI/R) underwent the same surgical procedures, except that the suture placed under the left coronary artery was not tied. At the end of reperfusion, the suture around the coronary artery was retied, and 2% Evans Blue dye was injected into the left ventricular cavity. The heart was quickly excised, and the I/R cardiac tissue was isolated and processed per the protocols described below.

### Determination of Cardiac Function, Myocardial Infarct Size, and Apoptosis

At the end of the 24h reperfusion period, the mice were re-anesthetized, and cardiac function was determined by noninvasive echocardiography (VisualSonics VeVo 2100 imaging system). Upon completion of the functional determination, the ligature around the coronary artery was retied, and MI size was determined by the Evans blue/TTC double-staining method (Tao et al., 2004; Tao et al., 2010). Myocardial apoptosis was determined within the entire I/R region *via* TUNEL staining and caspase-3 activity assays, as described previously (Tao et al., 2004; Tao et al., 2010).

### Determination of ATP Content

The ATP content of the myocardium was measured using an ATP bioluminescent assay kit (Beyotime, China).

### Isolation Mitochondria From Hearts

Hearts were rinsed in ice-cold medium A (120 mM NaCl, 20 mM HEPES, 2 mM MgCl2, 1 mM EGTA, and 5 g/l bovine serum albumin; pH 7.4) to remove any residual blood. Cardiac tissue was minced in ice-cold medium A and homogenized. The homogenate was centrifuged at 600×g for 10 min at 4°C. The supernatant fluid was subsequently centrifuged at 17,000×g for 10 min at 4°C. The pellet containing the mitochondria was re-suspended in medium A, and then centrifuged at 7,000×g for 10 min at 4°C. The pellet obtained after the last centrifugation was re-suspended in medium B (300 mM sucrose, 2 mM HEPES, 0.1 mM EGTA; pH 7.4) and re-centrifuged (3,500×g, 10 min, 4°C). The resulting pellet, which contained the heart mitochondria, was suspended in a small volume of medium B and stored at −70°C. The mitochondrial protein concentration was determined using the BCA Protein Assay kit (Beyotime, China) with bovine serum albumin (BSA) as a standard.

### Assays for Mitochondrial Enzyme Activities

Enzymatic activities of mitochondrial complexes I–V were measured as previously described (Yan et al., 2013). The mitochondrial marker enzyme CS was used as a reference.

### Determining Citrate Synthase (CS) Activity

CS activity of the myocardium and NRVMs was measured by using a commercially available citrate synthase activity assay kit (Sigma, USA).

### Western Blot Analysis

Proteins were separated on SDS-PAGE gels, transferred to PVDF (polyvinylidenedifluoride, Millipore), and incubated with antibodies against, Bax (1:1000, Abcam), Caspase-9(1:1000, Abcam), Bcl-2(1:1000, Abcam), Survivin(1:1000, Abcam), phospho-AMPK (Thr172, 1:1000, Cell Signaling Technology), AMPK (1:1,000, Cell Signaling Technology), and GAPDH (1:1,000, Santa Cruz Biotechnology) overnight at 4°C. After washing blots to remove excessive primary antibody binding, the blots were incubated for 1h with horseradish peroxidase (HRP)-conjugated secondary antibody (1:5,000, abgent). Antibody binding was detected using enhanced chemiluminescence (Millipore). Film was scanned with ChemiDocXRS (Bio-Rad Laboratory, Hercules, CA). Immunoblot band intensity was analyzed using Lab Image software.

### Statistics Analyses

Data were presented as mean ± SEM and the differences among three or more groups were analyzed by ANOVA. Western blot densities were analyzed with the Kruskal-Wallis test followed by a Dunn *post hoc* test. A P value <0.05 was considered statistically significant. All statistical tests were performed using GraphPad Prism software version 5.0 (GraphPad Software, San Diego, CA).

## Results

### Result 1: MI/R Injury Is Increased Markedly in Diabetic FNDC5^-/-^ Mice and Is Reduced by Irisin Treatment

**Figure 1:**
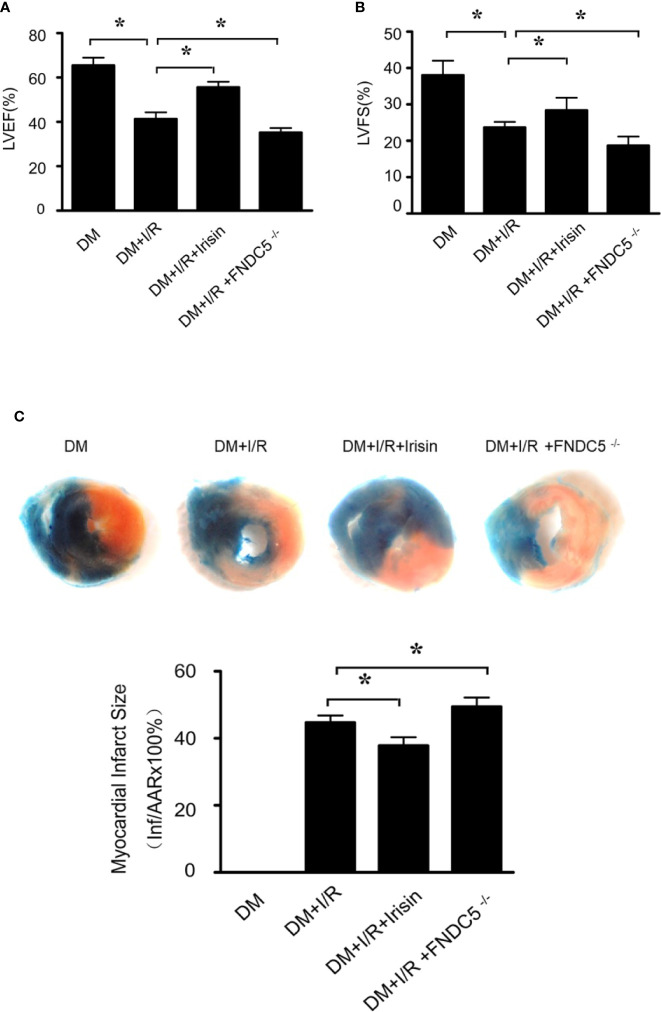
Myocardial ischemia-reperfusion (MI/R) injury is increased in diabetic FNDC5^-/-^ mice, while irisin treatment attenuates MI/R injury. **(A**, **B)**: left ventricular ejection fraction (LVEF; **A**) and left ventricular fraction shortening (LVFS; **B**) were determined by echocardiograph as indexes for cardiac function at the end of the 24-hour reperfusion. **(C)** myocardial infarct size was assessed by Evans blue/TTC double staining. All values are presented as mean ± SEM. n = 6–8/group. ^*^P < 0.05.

To investigate whether irisin plays a role in the protection of the heart from I/R, we induced 30min of ischemia followed by 24h of reperfusion in diabetic WT and FNDC5^-/-^ mice, and then determined infarct size and cardiac function. Compared with WT DM diabetic mice, diabetic FNDC5^-/-^ mice displayed significantly depressed cardiac function (**Figures 1A, B**) and enlarged infarct size (**Figure 1C**) after MI/R. These data suggest irisin plays a role in MI/R injury in diabetic mice. To further assess the potential protection by irisin, we administered irisin 30 min before ischemia. Irisin treatment significantly improved cardiac function (**Figures 1A, B**), reduced infarct size (**Figure 1C**) in diabetic mice. These findings suggested protective effects of irisin on the heart against I/R injury in diabetic mice.

### Result 2: Mitochondrial Dysfunction Is Increased Markedly in Diabetic FNDC5^-/-^ Mice and Is Repaired by Irisin Treatment

**Figure 2:**
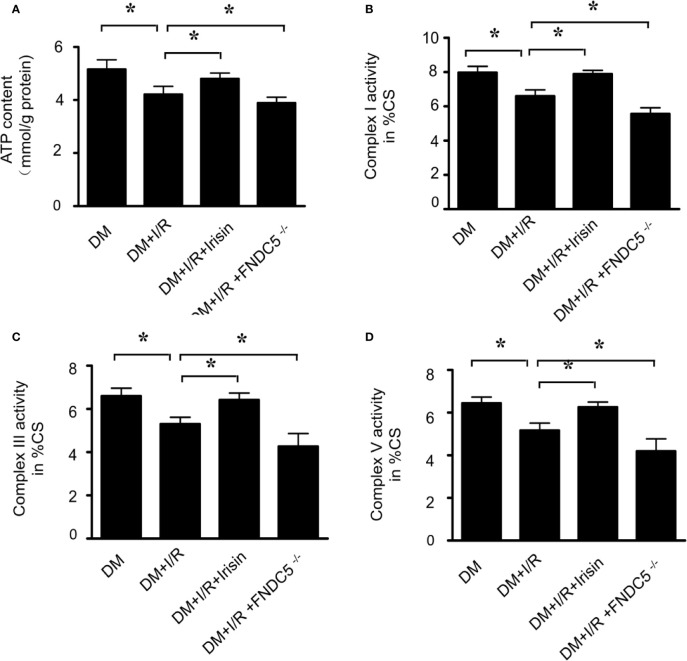
Mitochondrial function was more severely impaired in diabetic FNDC5^-/-^ mice received MI/R and Irisin reversed the mitochondrial dysfunction. **(A)** ATP production was measured to reflect the mitochondrial function. **(B–D)** Enzymatic activities of complexes I/III/V were determined in isolated mitochondria. All values are presented as mean ± SEM. n = 6–8/group. ^*^P < 0.05.

Mitochondrial energy metabolism is impaired in diabetic mice received MI/R. Activities of mitochondrial respiratory complexes I/III/V in diabetic hearts were significantly decreased compared with the normal ones (Yan et al., 2013). We detected the alterations of mitochondrial function in all the diabetic mice received MI/R. Compared with WT DM diabetic mice, ATP production (**Figure 2A**) was significantly reduced, and the activities of mitochondrial respiratory complex I/III/V (**Figures 2B–D**) were repressed in diabetic FNDC5^-/-^ mice, which suggests irisin plays a role in sustaining mitochondrial energy metabolism. Irisin treatment improved the ATP production (**Figure 2A**) and the activities of mitochondrial respiratory complex (**Figures 2B–D**). These findings suggested the mitochondria-protective effect of irisin in diabetic mice.

### Result 3: Mitochondrial Apoptosis Is Increased Markedly in Diabetic FNDC5^-/-^ Mice and Is Blocked by Irisin Treatment

**Figure 3:**
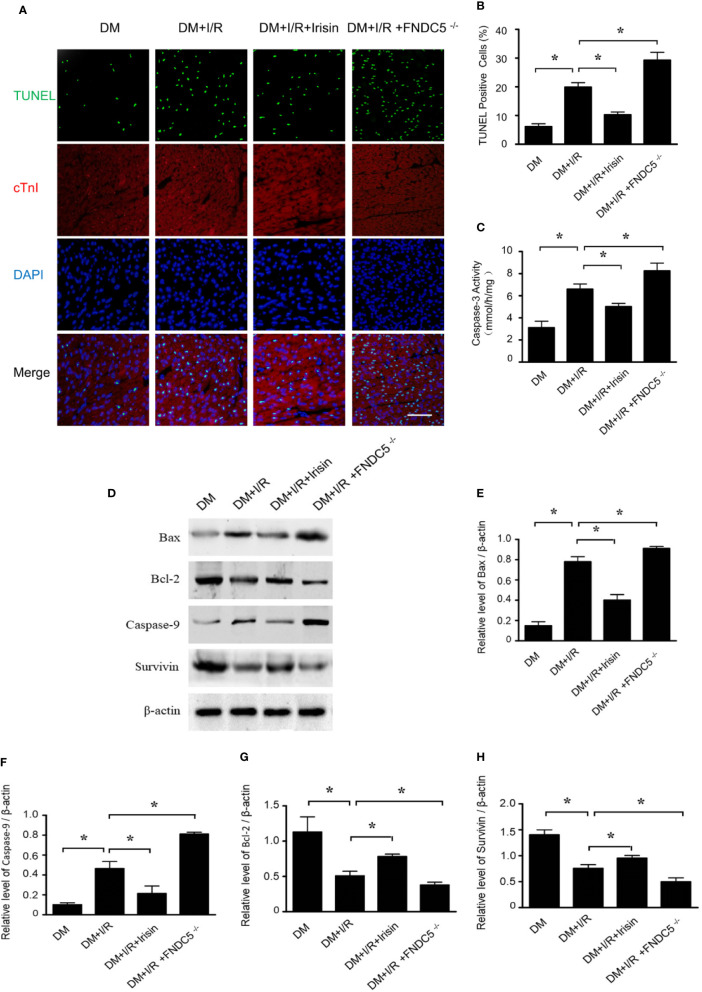
Apoptosis was increased in diabetic FNDC5^-/-^ mice compared with WT diabetic mice under MI/R injury and irisin inhibited mitochondrial apoptosis in cardiomyocyte in diabetic mice. Cardiomyocyte apoptosis was determined by TUNEL assay **(A, B)** and caspase-3 assay **(C)**. Mitochondrial apoptotic parameters **(D–G)**: **(E)** Bad, **(F)** Caspase-9, **(G)** Bcl-2, and **(H)** survivin) were determined *via* western blot analysis. All values are presented as mean ± SEM. n = 6–8/group. ^*^P < 0.05.

TUNEL staining (**Figures 3A, B**) and caspase-3 activity assay (**Figure 3C**) in I/R cardiac tissue demonstrated markedly increased cardiomyocyte apoptosis in diabetic FNDC5^-/-^ mice following MI/R. While irisin treatment significantly reduced cardiomyocyte apoptosis, as evidenced by reduced TUNEL staining (**Figures 3A, B**) and caspase-3 activity (**Figure 3C**) in diabetic mice. MI/R injury down-regulates the expression of pro-death parameters (Bax and caspase-9) and up-regulates pro-survival proteins (Bcl-2 and survivin) (Badalzadeh et al., 2015; Fan et al., 2020). To figure out the role of irisin on apoptosis under MI/R injury, protein detection was performed to verify the changes in apoptotic proteins (**Figures 3D–H**). Compared with WT DM mice, Bax (**Figure 3E**) and caspase-9 (**Figure 3F**) expression were elevated and Bcl-2 (**Figure 3G**) and survivin (**Figure 3H**) expression were inhibited markedly in diabetic FNDC5^-/-^ mice, which suggests irisin plays a role on mitochondrial apoptosis under MI/R injury in diabetic mice. Irisin treatment up-regulated the expression of Bcl-2 (**Figure 3G**) and survivin (**Figure 3H**), and down-regulated the expression of Bax (**Figure 3E**) and caspase-9 (**Figure 3F**). These findings exhibited the pro-survival effects of irisin on cardiomyocytes under MI/R in diabetic mice.

### Result 4: AMPK Pathway Was Impaired in All the Mice Received MI/R, Especially in Diabetic FNDC5^-/-^ Mice

**Figure 4:**
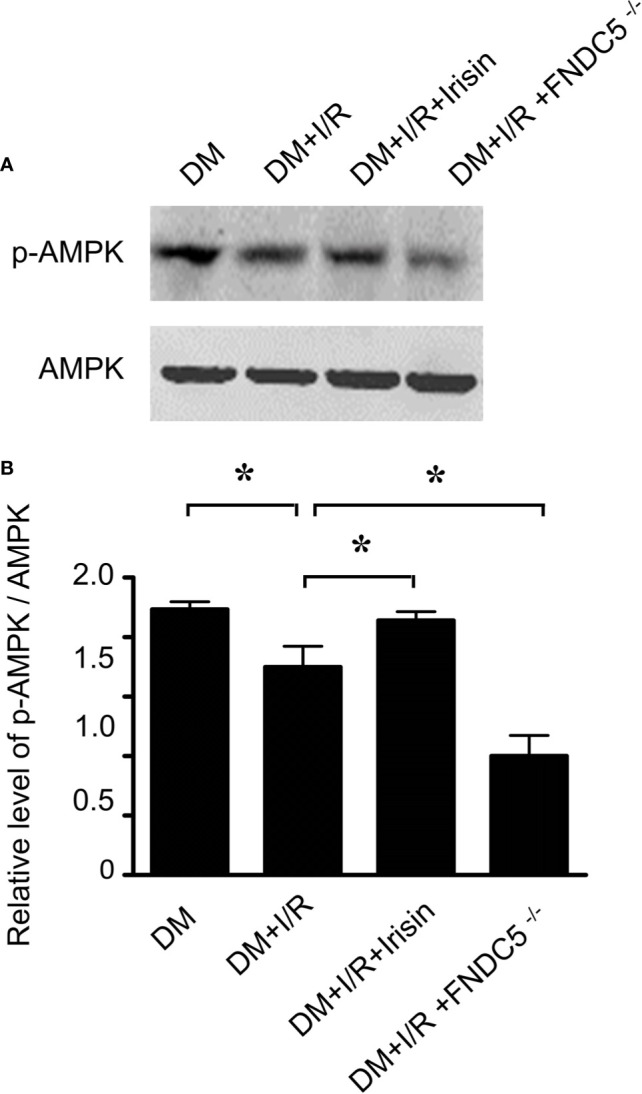
Irisin increases P-AMPK expression in diabetic mice received MI/R. P-AMPK and AMPK expression **(A, B)** was determined by Western blot analysis. All values are presented as mean ± SEM. n = 6–8/group. ^*^P < 0.05.

Irisin exerts its metabolic regulation largely through AMP-dependent protein kinase (AMPK). Irisin improves fatty acid oxidation and glucose utilization in type 2 diabetes by regulating the AMPK signaling pathway (Xin et al., 2016). However, the role of AMPK in irisin’s anti-apoptotic effect in I/R adult diabetic cardiomyocytes remains incompletely understood. Western blot was applied to observe the alterations of the AMPK pathway in diabetic mice received I/R. As indicated, I/R reduced the expression of P-AMPK in diabetic mice especially in diabetic FNDC5^-/-^ mice. Irisin reversed the activity of AMPK, by increasing the expression of P-AMPK (**Figures 4A, B**). These findings indicated that AMPK may play an essential role in irisin’s protective effect in I/R adult diabetic cardiomyocytes.

### Result 5: Knock-Out of Gene AMPK Inhibited the Effects of Irisin on Cardiac Function in Diabetic Mice

**Figure 5:**
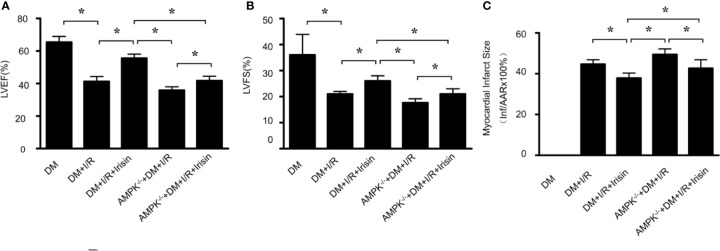
The effects of irisin on cardiac function in diabetic AMPK^-/-^ mice were inhibited. **(A**, **B)** left ventricular ejection fraction (LVEF; **A**) and left ventricular fraction shortening (LVFS; **B**) were determined by echocardiograph as indexes for cardiac function at the end of the 24-h reperfusion. **(C)** myocardial infarct size was assessed by Evans blue/TTC double staining. All values are presented as mean ± SEM. n = 6–8/group. ^*^P < 0.05.

To investigate whether AMPK plays a role in the cardio-protective effect of irisin from I/R, we induced 30 min of ischemia followed by 24h of reperfusion in diabetic WT and AMPK^-/-^ mice, and then determined infarct size and cardiac function. Compared with WT DM diabetic mice, diabetic AMPK^-/-^ mice displayed depressed cardiac function (**Figures 5A, B**) and enlarged infarct size (**Figure 5C**) after MI/R. The information suggests AMPK plays a role in MI/R injury in diabetic mice. To further assess the potential role of AMPK, we administered irisin 30 min before ischemia. All the effects of irisin on cardiac function (**Figures 5A, B**), infarct size (**Figure 5C**) in diabetic mice were significantly inhibited. These findings suggested the role of AMPK on irisin’s cardio-protective effects against I/R injury in diabetic mice.

### Result 6: Knock-Out of Gene AMPK Inhibited the Effects of Irisin on Mitochondrial Function in Diabetic Mice Received MI/R

**Figure 6:**
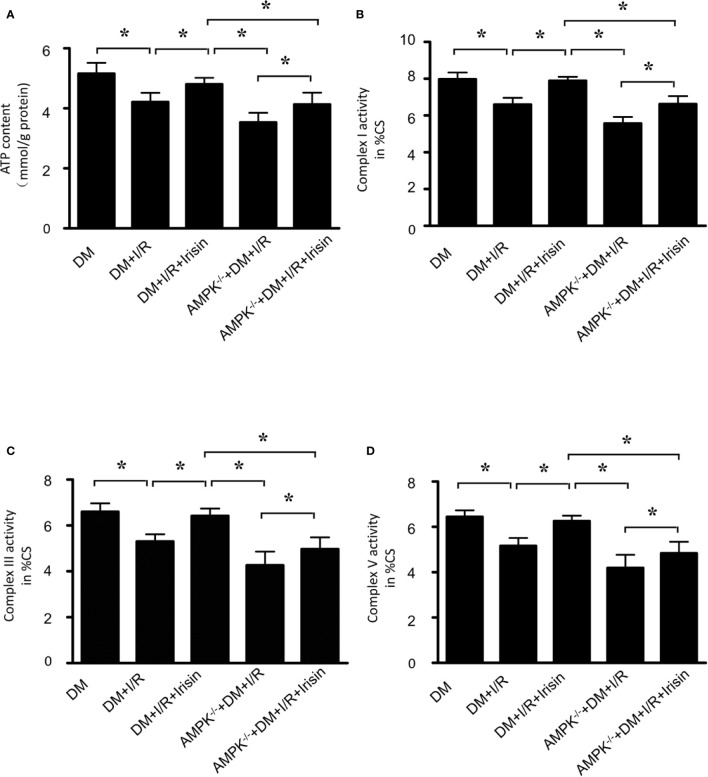
The effects of irisin on mitochondrial function in diabetic AMPK^-/-^ mice were inhibited. **(A)** ATP production was measured to reflect the mitochondrial function. **(B–D)** Enzymatic activities of complexes I/III/V were determined in isolated mitochondria. All values are presented as mean ± SEM. n = 6–8/group. ^*^P < 0.05.

Then we detected the alterations of mitochondrial function in diabetic AMPK^-/-^ mice received MI/R. Compared with WT DM diabetic mice, ATP production(**Figure 6A**) was significantly reduced, and the activities of mitochondrial respiratory complexes I/III/V (**Figures 6B–D**) were repressed in diabetic AMPK^-/-^ mice. These data suggests AMPK plays a role in irisin’s effects on mitochondrial energy metabolism, while irisin didn’t reverse the reduced ATP production (**Figure 6A**) and the activities of complexes I/III/V (**Figures 6B–D**). These findings suggested the essential role of AMPK on irisin’s mitochondria-protective effect in diabetic mice.

### Result 7: Knock-Out of Gene AMPK Inhibited the Effects of Irisin on Mitochondrial Apoptosis in Diabetic Mice Received MI/R

**Figure 7:**
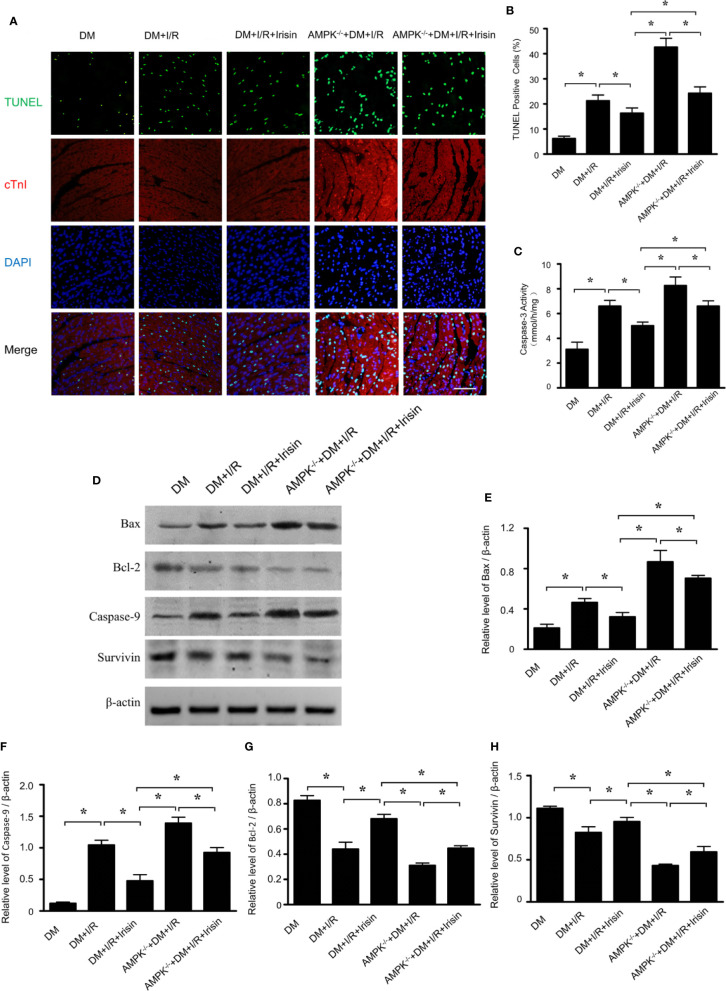
The effects of irisin on mitochondrial apoptosis in diabetic AMPK^-/-^ mice were inhibited. Cardiomyocyte apoptosis was determined by TUNEL assay **(A, B)** and caspase-3 assay **(C)**. Mitochondrial apoptotic parameters **(D–H)**: **(E)** Bad, **(G)** Caspase-9, **(G)** Bcl-2 and **(H)** survivin) were determined *via* western blot analysis. All values are presented as mean ± SEM. n = 6–8/group. ^*^P < 0.05.

TUNEL staining (**Figures 7A, B**) and caspase-3 activity assay (**Figure 5C**) in I/R cardiac tissue demonstrated markedly increased cardiomyocyte apoptosis in diabetic AMPK^-/-^ mice following MI/R. The effects of irisin on cardiomyocyte apoptosis, as evidenced by reduced TUNEL staining (**Figures 7A, B**) and caspase-3 activity (**Figure 7C**) in diabetic mice were significantly inhibited. To figure out the role of AMPK in irisin’s anti-apoptosis effects under MI/R injury, protein detection was performed to verify the changes in apoptotic proteins (**Figures 7D–H**). Compared with WT DM mice, Bax (**Figure 7E**) and caspase-9 (**Figure 7F**) expression were elevated and Bcl-2 (**Figure 7G**) and survivin (**Figure 7H**) expression were inhibited markedly in diabetic AMPK^-/-^ mice, which suggests AMPK plays a role on mitochondrial apoptosis under MI/R injury in diabetic mice. In diabetic AMPK^-/-^ mice, irisin treatment failed in up-regulating the expression of Bcl-2 (**Figure 7G**) and survivin (**Figure 7H**), and down-regulating the expression of Bax (**Figure 7E**) and caspase-9 (**Figure 7F**) as indicated in WT diabetic mice. These findings exhibited the essential role of AMPK in pro-survival effects of irisin on cardiomyocytes under MI/R in diabetic mice.

## Discussion

In the present study, we demonstrated that irisin reduced cardiac infarct size and improved post-I/R ventricular function, and irisin improved mitochondria function and suppressed mitochondrial apoptosis under MI/R injury in diabetic mice. Furthermore, knock-out of gene AMPK blocked irisin’s effects on cardiac function, mitochondria function and mitochondrial apoptosis in diabetic mice. Taken together, irisin attenuates MI/R injury, improves mitochondrial function and suppresses mitochondrial apoptosis through AMPK pathway in diabetic mice.

As a recently identified myokine, irisin mediates beneficial effects of exercise and affects energy, glucose and lipid metabolism, mainly controlled by PPARγ coactivatou1 alpha (PGC1α) (Bostrom et al., 2012). Irisin is produced in muscle as a cleavage product of the extracellular portion of type I membrane protein fibronectin type III domain containing 5 (FNDC5), which is proteolytically cleaved and secreted into circulation. Irisin regulates energy metabolism by promoting the browning of white adipose tissue and increasing the energy expenditure (Bostrom et al., 2012). Furthermore, irisin suppresses inflammation and oxidative stress induced by high circulation of glucose and obesity (Dulian et al., 2015; Lu et al., 2015; Zhu et al., 2015). Recent researches showed that irisin produces a cardioprotective effect (Wang et al., 2017; Fan et al., 2020). However, the necessity of irisin in MI/R injury in diabetic mice and its underlying mechanism remain unknown. In the present study, we established a mouse model of diabetes with MI/R by feeding FNDC5^-/-^ mice with high-fat diet. The lack of irisin induced significantly depressed cardiac function and enlarged infarct size after MI/R. Moreover, increased TUNEL staining and caspase-3 activity assay were also observed in diabetic FNDC5^-/-^ mice received MI/R. Pretreatment of irisin in diabetic mice with MI/R attenuated myocardial infarct size and improved cardiac function, as well as reduced myocardial cell apoptosis showed by reduced TUNEL staining and caspase-3 activity. Our results provided direct evidence supporting that irisin plays a beneficial role in MI/R injury in diabetes.

Mitochondria play an important role in diabetes, cardioprotection, and I/R injury (Sharafati-Chaleshtori et al., 2017). Mitochondrion has been reported to be a key target of cardiomyocyte death mediated by hypoxia/re-oxygenation injury in the presence of hyperglycemia stress. Mitochondrial dysfunction manifested as decreased ATP content, citrate synthase (CS) activity and complexes I/III/V activities, occurs in diabetic hearts (Yan et al., 2013). Considering the role of irisin in energy homeostasis, then we investigated whether irsin repaired mitochondria dysfunction. The lack of irisin induced significantly decrease of ATP production and the activities of mitochondrial respiratory complexes I/III/V, compared with diabetic WT mice with MI/R injury which suggested irisin’s essential role in sustaining mitochondrial energy metabolism in diabetic mice with MI/R injury. Pretreatment of irisin in diabetic mice with MI/R improved the ATP production and the activities of mitochondrial respiratory complexes. Therefore, irisin repairs mitochondrial dysfunction in diabetic mice with MI/R.

Apoptosis is an important cause of diabetic cardiomyopathy. Diabetes impairs the anti-apoptotic intracellular signaling cascades involved in myocardial protection. Anti-apoptotic parameters including Bcl-2, Bcl-xl, Bcl-w, MCl-1, and A_1_/BFI-1, inhibit release of mitochondrial apoptogenic factors following cell death triggers. Pro-apoptotic parameters including BID, BAX, BAK, BAD and ect, play important roles in cell death during reperfusion injury (Brady et al., 2006). Over-expression of Bcl-2 reduces IR injury (Chen et al., 2001). Bcl-2 decreases acidification and ATP deprivation during ischemia (Dong et al., 2003; Imahashi et al., 2004; Hu et al., 2013). Furthermore, it is also reported that the increases in the proportion of Bcl-2 to BAX can prevent myocardial apoptosis progress induced by ischemia and reperfusion (Dong et al., 2003; Hu et al., 2013). In the present study, we demonstrated that the lack of irisin increased apoptosis in diabetic mice with MI/R injury compared with diabetic WT mice, evidenced by elevated expression of pro-apoptotic parameters (Bax and caspase-9) and depressed expression of anti-apoptotic parameters (Bcl-2 and survivin). While irisin treatment inhibited the increase of the expression of Bax and caspase-9, and blocked the depression of the expression of Bcl-2 and survivin, which suggests that irisin blocks mitochondrial apoptosis pathway in cardiomyocyte in diabetic mice with MI/R.

AMP-activated protein kinase (AMPK) is a serine–threonine kinase that acts as an energy sensor and can be activated by hypoxia stress. When cellular energy is depleted, AMPK activation down-regulates ATP utilizing pathways and up-regulates ATP-generating pathways, which restores energy homeostasis (Carling, 2004). Modulation of AMPK activity in the diabetic heart improves cardiac function and attenuates MI/R injury. During I/R injury, AMPK is activated, which stimulates glucose uptake and glycolysis during ischemia so to supply the ATP for cardiac function (Paiva et al., 2011; Hermann et al., 2012). AMPK activation inhibits the fission-mediated mitochondrial stress in reperfusion-mediated mitochondrial fission in cardiomyocytes. Reduced myocardial AMPK activity leads to impaired mitochondrial function (AbdElmonem Elbassuoni, 2014). In diabetic cardiomyopathy, activation of AMPK retards the complications of diabetes.

Irisin improves fatty acid oxidation and glucose utilization in type 2 diabetes by regulating the AMPK signaling pathway (Xin et al., 2016). Irisin inhibits pancreatic cancer growth *via* activating AMPK pathway (Liu et al., 2018). Irisin modulate cardiomyocyte morphology through AMPK in pressure overload-induced cardiac hypertrophy (Li et al., 2018). Besides, irisin improves endothelial dysfunction *via* AMPK pathway (Zhu et al., 2015). In diabetic FNDC5^-/-^ mice, we observed a lower level of P-AMPK than that in diabetic WT mice, while irisin treatment reversed the activity of AMPK, by increasing the expression of P-AMPK, suggesting AMPK may mediates the protective effects of irisin on diabetic heart. Then we established a mouse model of diabetes with MI/R by feeding FNDC5^-/-^ mice with high-fat diet. All the protective effects of irisin on cardiac function, infarct size, and cardiomyocyte apoptosis, as evidenced by TUNEL staining and caspase-3 activity in diabetic mice were significantly inhibited. Knock-out of gene AMPK inhibited the effects of irisin on mitochondrial function and mitochondrial apoptosis.

In summary, we provided evidence showing that irisin attenuates myocardial ischemia/reperfusion injury and improves mitochondrial function through AMPK pathway in diabetic mice. Our results indicate that targeting irisin is a promising approach for the treatment of MI/R injury in diabetes.

## Data Availability Statement

The datasets presented in this study can be found in online repositories. The names of the repository/repositories and accession number(s) can be found in the article/supplementary material.

## Ethics Statement

The animal study was reviewed and approved by ethics committee in Second Artillery General Hospital of Chinese People’s Liberation Army.

## Author Contributions

The work presented here was carried out in collaboration between all authors. LT and ZJ defined the research theme and revised the manuscript critically. CX, ZZ, and GG designed methods and experiments, carried out the laboratory experiments, and wrote the paper. LD, CY, CW, YJL, YG, XY, LJZ, LNZ, and YL collected and analyzed the data, interpreted the results.

## Conflict of Interest

The authors declare that the research was conducted in the absence of any commercial or financial relationships that could be construed as a potential conflict of interest.
